# Anticancer Molecular Mechanisms of Resveratrol

**DOI:** 10.3389/fnut.2016.00008

**Published:** 2016-04-12

**Authors:** Elena M. Varoni, Alfredo Fabrizio Lo Faro, Javad Sharifi-Rad, Marcello Iriti

**Affiliations:** ^1^Dipartimento di Scienze Biomediche, Chirurgiche ed Odontoiatriche, Università degli Studi di Milano, Milan, Italy; ^2^Zabol Medicinal Plants Research Center, Zabol University of Medical Sciences, Zabol, Iran; ^3^Department of Pharmacognosy, Faculty of Pharmacy, Zabol University of Medical Sciences, Zabol, Iran; ^4^Dipartimento di Scienze Agrarie e Ambientali, Università degli Studi di Milano, Milan, Italy

**Keywords:** stilbenes, cancer chemoprevention, polyphenols, nutritional therapy, multi-target agents, bioavailability

## Abstract

Resveratrol is a pleiotropic phytochemical belonging to the stilbene family. Though it is only significantly present in grape products, a huge amount of preclinical studies investigated its anticancer properties in a plethora of cellular and animal models. Molecular mechanisms of resveratrol involved signaling pathways related to extracellular growth factors and receptor tyrosine kinases; formation of multiprotein complexes and cell metabolism; cell proliferation and genome instability; cytoplasmic tyrosine kinase signaling (cytokine, integrin, and developmental pathways); signal transduction by the transforming growth factor-β super-family; apoptosis and inflammation; and immune surveillance and hormone signaling. Resveratrol also showed a promising role to counteract multidrug resistance: in adjuvant therapy, associated with 5-fluoruracyl and cisplatin, resveratrol had additive and/or synergistic effects increasing the chemosensitization of cancer cells. Resveratrol, by acting on diverse mechanisms simultaneously, has been emphasized as a promising, multi-target, anticancer agent, relevant in both cancer prevention and treatment.

## Introduction

Stilbenes are secondary metabolites produced by plants in response to stressful conditions, particularly fungal infection and UV radiation. Resveratrol, naturally occurring in some plant foods, but especially contained in grapes and red wine, is the most investigated and well-known member of this class of compounds. Being already in use in clinical settings because of the documented cancer and chemopreventive activities (1), it recently displayed a high impact in oncology, identifiable by checking the number of clinical trials, registered and ongoing, on international databases, such as US and EU clinical trial registers (Table 1). Moreover, epidemiological data support as the consumption of resveratrol is associated with the inhibition of several chronic disorders, including cancer (2). In particular, resveratrol-rich food intake has been reported to produce a significant decrease in the incidence of skin and breast cancers as well as in the progression of lung adenocarcinoma (3, 4).

**Table 1:** **Clinical trials on resveratrol and cancer recorded on US and EU registers**.

Title (identifier number)	Condition(s)
**www.clinicaltrials.gov**	
Resveratrol for Patients with Colon Cancer (NCT00256334)	Colon cancer
Resveratrol in Treating Patients with Colorectal Cancer that Can Be Removed by Surgery (NCT00433576)	Adenocarcinoma of the colon/rectum
A Biological Study of Resveratrol’s Effects on Notch-1 Signaling in Subjects with Low-Grade Gastrointestinal Tumors (NCT01476592)	Neuroendocrine tumor
Phase I Biomarker Study of Dietary Grape-Derived Low Dose Resveratrol for Colon Cancer Prevention (NCT00578396)	Colon cancer
Resveratrol and Human Hepatocyte Function in Cancer (NCT02261844)	Liver cancer
A Clinical Study to Assess the Safety, Pharmacokinetics, and Pharmacodynamics of SRT501 in Subjects with Colorectal Cancer and Hepatic Metastases (NCT00920803)	Colon-rectal cancer
UMCC 2003-064 Resveratrol in Preventing Cancer in Healthy Participants (NCT00098969)	Unspecified adult solid tumors
A Clinical Study to Assess the Safety and Activity of SRT501 Alone or in Combination with Bortezomib in Patients with Multiple Myeloma (NCT00920556)	Multiple myeloma
**www.clinicaltrialsregister.eu**	
Prostate Phytochemical & PUFA Intervention (EudraCT Number: 2006-006679-18)	Localized prostate cancer

This narrative review aims to summarize the current body of evidence, within the last 10 years, about the anticancer and chemoprevenive molecular mechanisms of resveratrol. In October 2015, we consulted PubMed and EMBASE databases for extensive search of articles on this topic. Multiple molecular targets, related to the different carcinogenesis pathways, were analyzed focusing on preclinical evidence, necessary both to screen molecules up to support *in vitro* and, then, *in vivo* efficacy and to provide clues on their molecular basis of activity (5). Particular emphasis was provided to the ability of resveratrol in reducing the risk of multidrug resistance (MDR), *via* multiple cellular targets involved in carcinogenesis and chemo/radioresistance, which mediate its synergy with the chemotherapeutics (6). In this direction, a more ­punctual ­comprehension of resveratrol mechanism(s) of action can promote the development of novel, multi-target cancer therapies, in order to improve drug efficacy and MDR elusion.

## Chemistry and Bioavailability of Resveratrol

**Figure 1:**
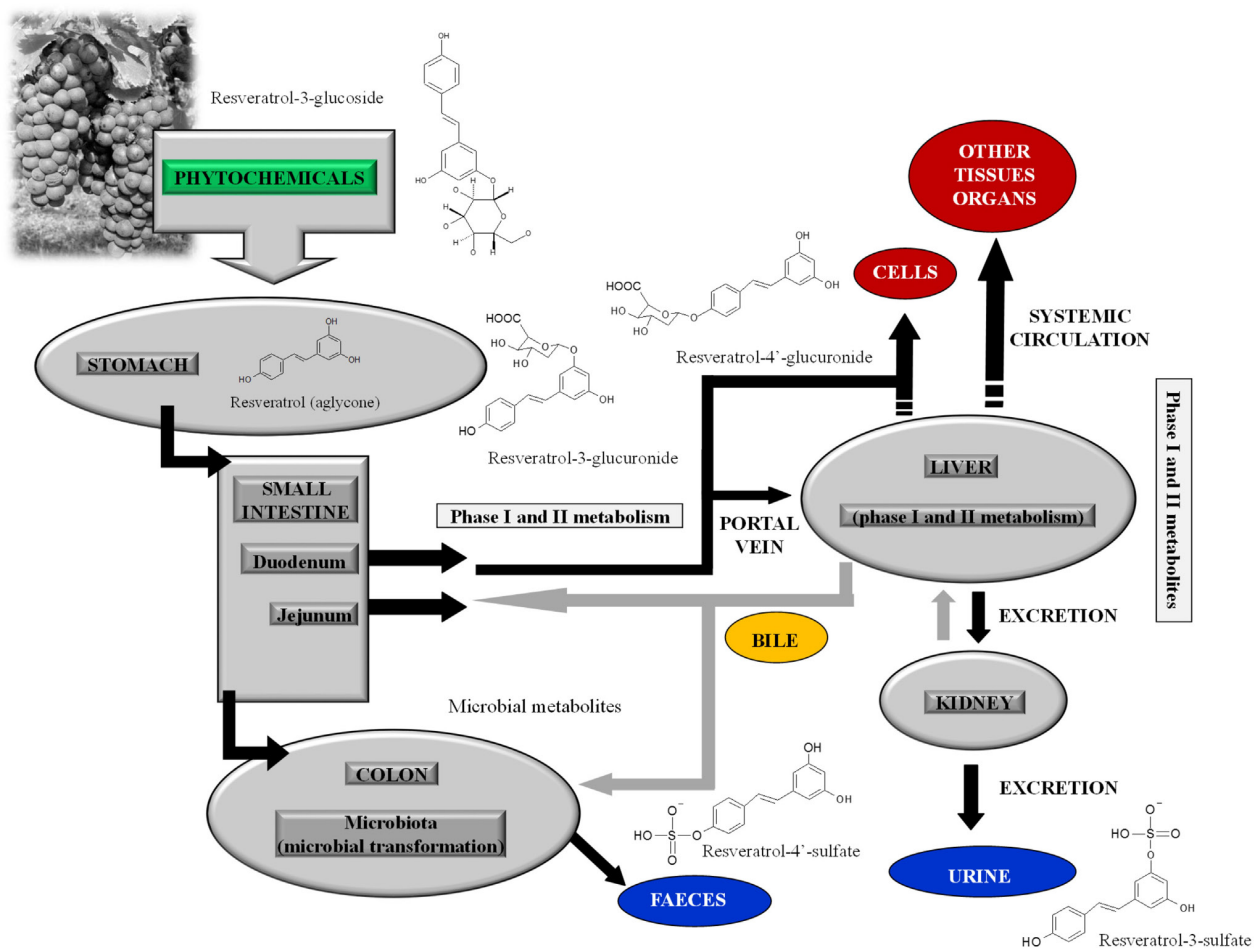
**Metabolism of resveratrol-3-glucoside (piceid), the main metabolite of resveratrol present in plant foods, in human gastrointestinal tract; after piceid deconjugation, resveratrol-3-glucuronide, resveratrol-4′-glucuronide, resveratrol-3-sulfate, and resveratrol-4′-sulfate are the main phase II metabolites of resveratrol aglycone**.

Resveratrol (*trans*-3,4′,5-trihydroxystilbene) is a non-flavonoid polyphenol belonging to the stilbenes. In plants, the molecule exists in two isomers, *trans*-resveratrol and *cis*-resveratrol, and their glucosides, *trans*-piceid and *cis*-piceid. The unconjugated (aglycone) *trans*-isomer is the most studied, *in vitro*, at concentrations largely exceeding those found in the human circulatory system after dietary intake. A recent study suggested that to accomplish the same concentrations used *in vitro*, 111 glasses of wine need to be consumed daily to reach the Bench Mark Dose (7). Moreover, resveratrol possesses a low bioavailability: it is rapidly metabolized in intestine and liver by phase II enzymes, and the end products of this metabolism are mainly glucuronide and sulfate derivatives (8) (Figure 1).

In particular, physiological levels of resveratrol-3-*O*-­glucuronide, resveratrol-4′-*O*-glucuronide, and resveratrol-3-*O*-sulfate have been recently tested and showed inefficacy against Jurkat T leukemia cells (9). None of these metabolites arrested or impaired cell proliferation; the unique evidence was the induction of mitochondrial membrane depolarization by resveratrol-3-*O*-sulfate, devoid of any consequence on cell death (9).

## Targeted Molecular Pathways

**Figure 2:**
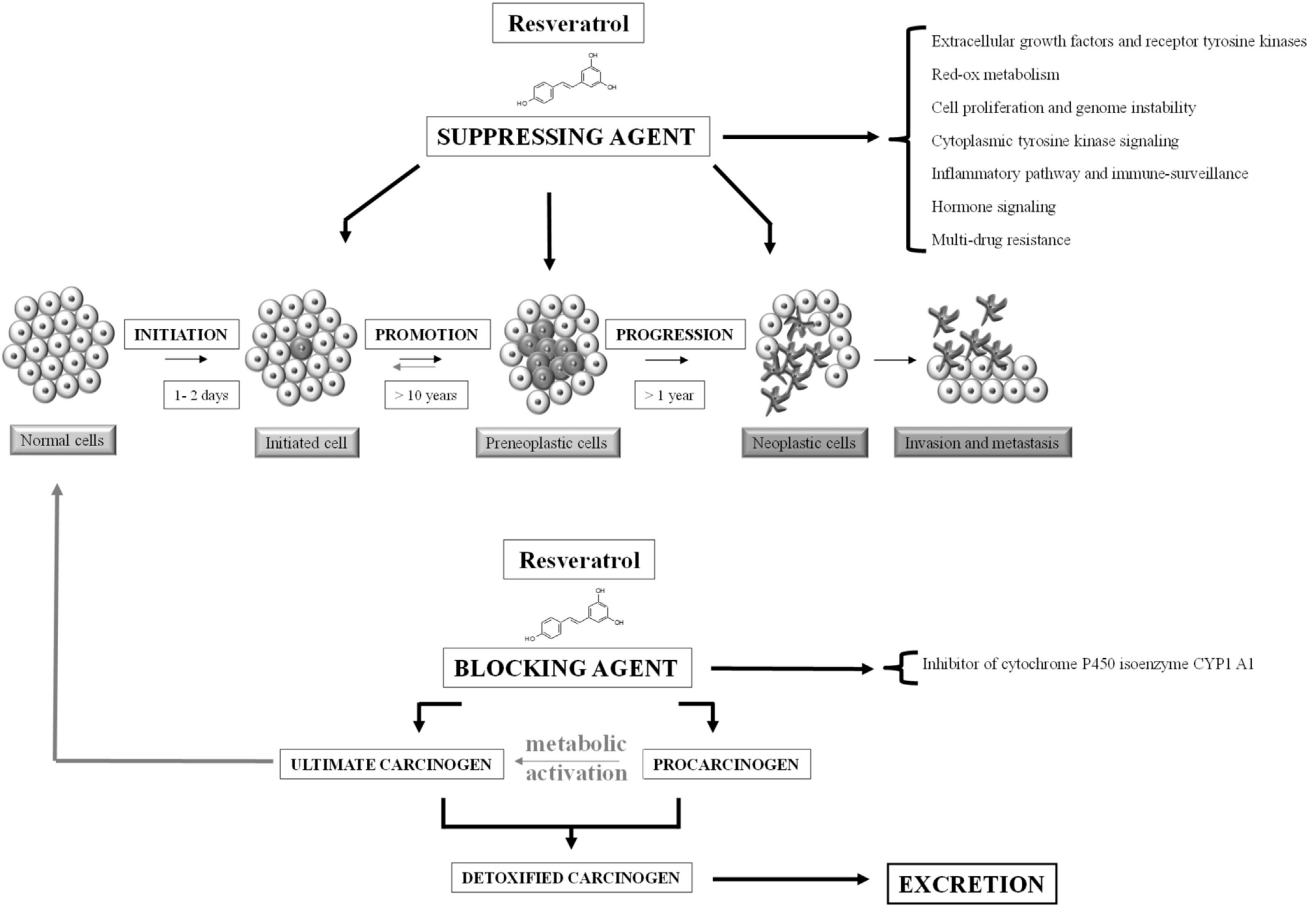
**Carcinogenesis and cancer chemoprevention**. Neoplastic process consists of three distinct, temporally ordered and linked stages: initiation, promotion, and progression. Chemopreventive agents are of two types: suppressing agents that inhibit the malignant transformation of initiated/preneoplastic cells and blocking agents that prevent the metabolic activation of procarcinogens [adapted from Ref. (14)].

**Figure 3:**
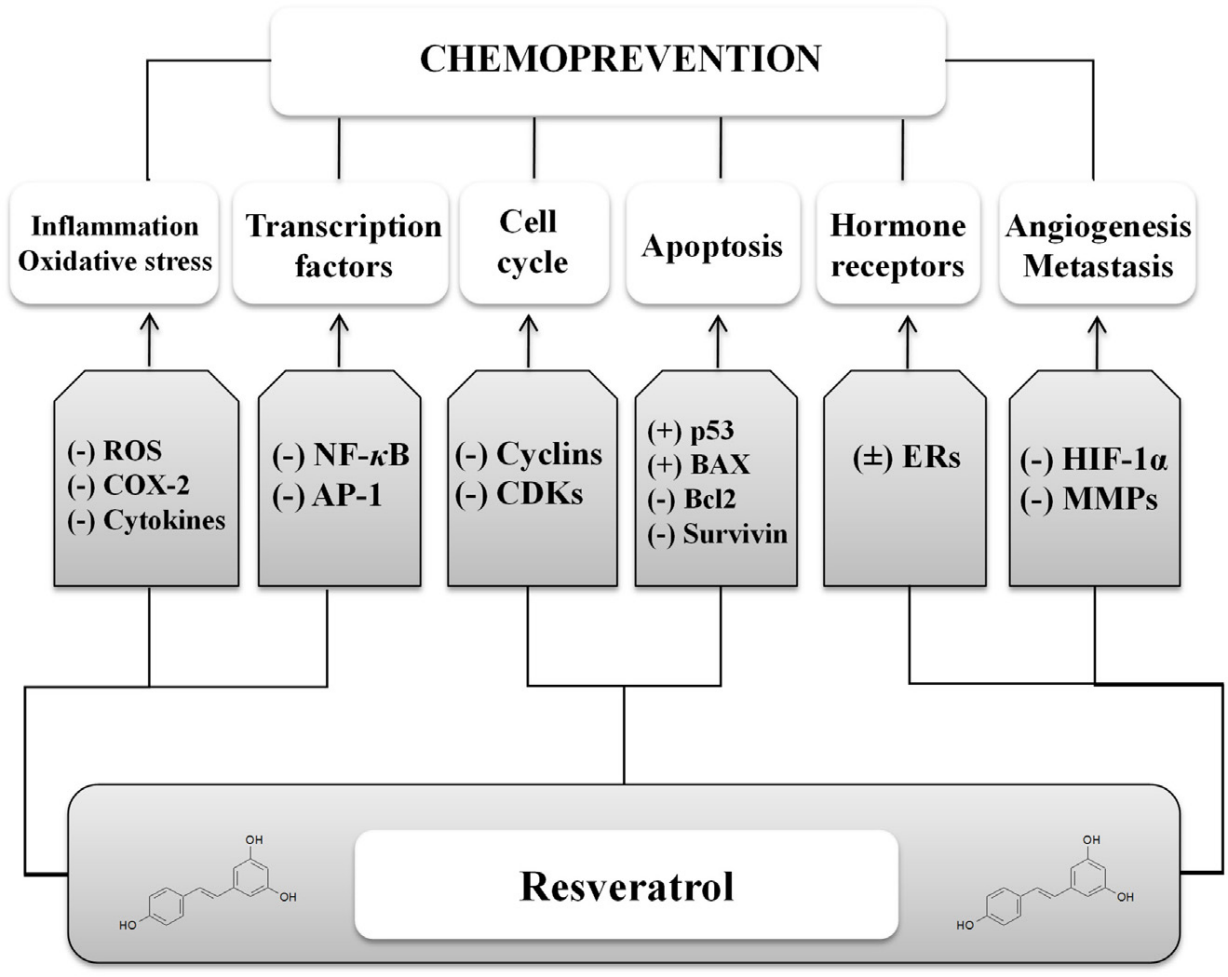
**Mechanisms involved in the anticancer activity of resveratrol**. A number of cell processes are targeted by resveratrol, by upregulating (+) or downregulating (−) different pathways. ROS, reactive oxygen species; COX, cyclooxygenase; NFκB, nuclear factor κB; AP-1, activator protein 1; CDKs, cyclin-dependent kinases; BAX, B cell lymphoma-associated X; Bcl2, B cell lymphoma 2; ERs, estrogen receptors; HIF-1α, hypoxia-induced factor 1α; MMPs, matrix metalloproteinases.

Preclinical data support phytochemicals interaction with many molecular and biochemical targets involved in chemical carcinogenesis, a complex three-stage process from cancer initiation to promotion and progression, up to invasion and metastasis (Figure 2) (10). In case of resveratrol, many chemopreventive and chemotherapeutic mechanisms to prevent, arrest, or reverse carcinogenesis stages have been investigated. Following mechanisms shared among several phytochemicals, resveratrol can act as suppressive agent on several impaired signaling pathways, acquiring the role of functionally pleiotropic agent, which expresses its activity on multiple targets in cancer cells with limited toxicity toward normal cells (Figure 3). Among cellular changes, the most important ones can be identified in accelerated transition of cells through cell cycle checkpoints with abnormal cell proliferation, genome instability, abnormal response to signals or other stimulators of programed cell death, uncontrolled neoangiogenesis, increased oxidative stress, overproduction of growth regulatory hormones, and alteration in host immune responses. Further contribution comes from the antioxidant, anti-inflammatory and immunomodulatory activities, reducing damage induced by oxidative stress (DNA damage, protein oxidation, and lipid peroxidation) and increasing immune oncosurveillance (10, 11). Resveratrol can also act as blocking agent by stopping the transformation of procarcinogen to carcinogen, since it inhibits the monooxygenase cytochrome P450 isoenzyme CYP1 A1, the enzyme deputed to the liver metabolism of xenobiotics (12, 13).

Along these lines, recent efforts in cancer therapeutics are directed against aberrant signal transduction pathways related to a diverse range of conditions; resveratrol appears to display multiple, contextual targeting activities by specific interaction with different biomolecules (15). Table 2 summaries the multi-target anti-cancer activities of resveratrol and related preclinical evidences.

**Table 2:** **Molecular mechanism of resveratrol and derivatives anticancer activity**.

Target in carcinogenetic processes	Cancer cells	Molecular pathways	Reference
Extracellular growth factors and receptor tyrosine kinases	Melanoma	EGF-R/EGF, TGF-β, Her-2, p53, TSP1, and VEGF	(16)
	Human umbilical vein endothelial cells	NO/eNOS, NO/cGMP/PKG, IAP1 and 2, livin, and XIAP	(17)
	Ovarian carcinoma (Her-2^+^ and −)		(18)
			(19)
Formation of multiprotein complexes, signal transmission and cell metabolism (red–ox)	Prostate carcinoma	PTEN/AKT, mTOR, FOXO, DNA damage leukocytic, red–ox imbalance (enzymatic and non-enxymatic mechanisms), SOD and CAT, DNA glycosylase I	(20)
	Melanoma		(21)
	Colon carcinoma		(22)
	Ovary carcinoma		(23)
	T cell leukemia Jurkat		(24)
	Lung adenocarcinoma (+As_2_O_3_)		(25)
Biological outcomes of growth factor signaling: cell proliferation and genome instability	Oral squamous cell carcinoma	Cell cycle arrest at G2/M and at G1/S transition, cyclin A2 and cyclin B1, cyclin D1, cyclin D2 and cyclin E, CDK inhibitor-cyclin-CDK machinery, WAF1/p21, CDK2, CDK4, and CDK6	(26)
	Human epidermoid carcinoma		(27)
	Anaplastic thyroid carcinoma		(28)
	Colon carcinoma	A3 adenosine receptors, lncRNA (AK001796), TOPO2, H2AX	(31)
	Lung adenocarcinoma		(32)
	Gliobastoma		(33)
	Breast carcinoma		(34)
	Non-melanoma skin cancer (+5-FLU)		(35)
	*Lung adenocarcioma*^a^	*BRCA1, cyclin B1, pRb, and p21*	(36, 37)
	*Osteosarcoma*	TOPO2	(38)
	*Colon carcinoma (*+*DOX)*		(39)
Cytoplasmic tyrosine kinase signaling	Human embryonic kidney cell line	NF-κB, IKKα, IKK-γ, NEMO, p65, TNF-α, Ad.Egr-TNF, Egr-1 promoter, AP-1, AP-2 and cAMP, CREB, MMP-2, and miR-328	(42)
	Breast carcinoma		(44)
Cytokine signaling	Pancreas adenocarcinoma		(44)
	*Human Embryonic Kidney cell line*	*SIRT1*	(35)
			(43)
			(50)
			(51)
Integrin signaling	Osteosarcoma	JNK, p38 MAPK, GADD45α, ERK/JNK-ElK-1, CArG, Egr-1 AP-1/JunD, MMP-1, iNOS, α-MSH β-catenin, c-kit, and MITF	(52, 53)
	Lung adenocarcinoma		(54)
	Pancreas adenocarcinoma	EMT: MMP-1, PI-3K/Akt/NF-κB, E-cadherin, N-cadherin, vimentin, MMP-2 and MMP-9, phospho-Akt and phospho-NF-κB, TGF-β, α5β1 integrins, hyaluronic acid, Snail, E-cadherin, and N-cadherin	(56)
	Colon–rectum adenocarcinoma		(27)
	Gastric carcinoma		(33)
			(53)
			(55)
			(56)
Developmental signaling pathways	Colon–rectum carcinoma	Wnt: Wnt2; Notch: TTF1, TTF2, Pax8	(57)
	Ovarian carcinoma	Notch: Notch1, NIS, Notch1 activation-dependent p53	(58)
	Gastric carcinoma	STAT3: Erk1/2(MAPK), pSTAT1	(59)
	Cervix adenocarcinoma and squamous cell carcinoma	*Hh*: GL1, Ihh, Ptch and Smo, PKC α and δ	(16)
			(53)
	Thyroid carcinoma		(60)
	Glioblastoma		
Signal transduction by the transforming growth factor	Melanoma	TGF-β, TGF-β1/Smads	(17)
	Pancreas adenocarcinoma		(23)
	Colon–rectum carcinoma		(24)

	Prostate carcinoma		(27)
Apoptotic pathways	Nasopharynx carcinoma		(29)
	Fibrosarcoma		(30)
	Liver carcinoma		(31)
	Hepatoma	Bcl-2, Bcl-X(L), survivin, Bax, Bak, PUMA, Noxa, Bim, TRAIL-R1/DR4 and TRAIL-R2/DR5, cytochrome *c*, Smac/DIABLO, and AIF	(38)
	Human epidermoid carcinoma		(43)
	Ovarian carcinoma	CASP (3, 8, and 9), PARP-1, myeloid cell leukemia 1 phospho-ERK and phospho-p38 expression	(61, 62)
	Colon–rectum carcinoma cells		(64)
	Colon–rectum carcinoma (DOX)	Atg5, Atg7, Atg9, and Atg12 proteins	(65)
	Lung (+As_2_O_3_)	p53, pRb-E2F/DP, Fas, FasL	(29)
	Non-melanoma skin cancer cell (+CIS)	*Fas, Fas ligand, extracellular-signal-regulated kinases (ERK) ½, and p38 expression*	
	Liver carcinoma		
		*Fas/APO-1*	
Inflammatory pathway	Colon–rectum carcinoma	COX-2, NF-κB, AP-1, IL-6, IL-8, TNF-α, ICAM-1, MCP-1, miRNAs, and PPAR	(15, 66, 67)
			(68)
			(69)
Immune surveillance	Splenocytes	IFN-γ, CD206, CD204, IL-10, TGF-β, EGF, MMP-9, IL-6, and IL-12	(71)
	Macrophages and tumor-associated macrophages (TAM)		
Hormone signaling	Breast carcinoma	Tif2, ERα, p53, and MAPK	(70, 73)
Multidrug resistance	Colon–rectum carcinoma (+5-FLU)	Desmosomes, gap- and tight junctions (claudin-2) and adhesion molecules (E-cadherin – EMT)	(23)
	Non-small cell lung cancer cells (+5-FLU)	CASP 3, MMP-9	(32)
	Non-melanoma skin cancer cell (+CIS)	NF-κB, cytochrome *c*, Bcl-2 and Bax, Fas and FasL	(38)
	Lung adenocarcioma (+As_2_O_3_)	Oxidative stress, cycle arrest at G1/S phase	(43)
	*Colon carcinoma (*+*DOX)*	*TOPO2*	(64)
			(38)

*^a^In italics are reported negative results (lack of anti-cancer activity on the selected target)*.

### Extracellular Growth Factors and Receptor Tyrosine Kinases

Antigrowth signaling activity is among the major targets of phytochemicals. Resveratrol specifically targets epidermal growth factor (EGF) and related receptor (EGF-R), a transmembrane tyrosine kinase activated by ligands, and the transforming growth factor-beta (TGF-β). EGF-R promotes cell growth and proliferation, and it is typically over-expressed in aggressive tumor phenotypes (16). Acting on EGF-R pathway, resveratrol and two of its derivatives, acetyl-resveratrol and polydatin, displayed the dose-dependent antigrowth effects against 3D cell aggregates of the EGF-R/Her-2-positive and -negative ovarian cancer cell lines, although *via* different signaling molecules (17). When tested on the positive ovarian cell line and at high concentrations, in particular, resveratrol and polydatin (but not acetyl-resveratrol) significantly reduced the phosphorylation of Her-2 and EGF-R, and decreased the expression of extracellular-signal-regulated kinases (ERK) and vascular endothelial growth factor (VEGF) (17). In agreement with these findings, Strickland and al. reported the inhibition of VEGF production, contextually to the amplified expression of p53 and matrix protein TSP1, testing resveratrol on a coculture of vascular endothelial and melanoma cells (18).

To better clarify the role of the molecule on the endothelial function and the pro- and antiangiogenic activities, the nitric oxide (NO) pathway was also investigated. In human umbilical vein endothelial cells (HUVECs), resveratrol raised NO production *via* increasing expression and activation of endothelial NO synthase (e-NOS), particularly acting on endogenous downstream cyclic guanidin monophosphate/protein kinase G (NO/cGMP/PKG) pathway and downstream cell-survival proteins [baculoviral inhibitor of apoptosis proteins (IAPs)] (19). Resveratrol, at high concentrations, inhibited HUVEC tube formation and cell migration and invasion, indices of neo-angiogenesis; it also suppressed endogenous PKG kinase activity and decreased the expression of four cell-survival proteins, i.e., IAP repeat-containing protein 2 and 3 (c-IAP1 and 2), livin, and the X-linked inhibitor of apoptosis (XIAP), which is E3 ubiquitin protein ligase (19). At low concentrations, in contrast, it stimulated cell proliferation, protecting HUVECs against apoptosis (19).

These findings support the function of resveratrol as suppressive agent able to block all carcinogenetic stages mediated by over-expression of growth factors and receptor tyrosine kinases. Acting particularly on EGF, resveratrol suppresses initiation, promotion, and progression of carcinogenesis while reducing VEGF expression as well as promoting NOS activity; it can prevent the formation of more aggressive tumor phenotypes, reducing neo-angiogenesis and the risk of metastasis and cancer-related tissue hypoxia.

### Formation of Multiprotein Complexes, Signal Transmission, and Cell Metabolism (Red–Ox)

Activated receptor protein tyrosine kinases (RPTKs) produce networks of signaling molecules, which consist of both preformed and rapidly associating protein complexes that transmit information throughout the cell. Growth factor receptors can activate, in turn, phosphoinositide signaling, where phospholipids of cell membrane contribute to signal propagation *via* two main mechanisms: (i) serving as precursor of the second messengers diacylglycerol (DAG), phosphatidylinositol 3-kinase (PI3K), and Ca^2+^ or (ii) binding to signal proteins containing specific phosphoinositide-binding modules.

Among the others, protein kinase B (PKB, also named AKT), rat sarcoma (RAS), and rapidly accelerated fibrosarcoma (RAF) are three of the most important serine/threonine-specific protein kinases, with a pivotal regulatory role in signaling cascade. AKT is activated by conformational changes evoked by phospholipid binding, and it phosphorylates two major downstream kinases, i.e., phosphoinositide-dependent kinase 1 (PDK-1) and the complex named transducer of regulated “cAMP response element-binding (CREB)” (TORC)-2; the latter includes serine threonine kinase mammalian target of rapamycin (mTOR). AKT activation regulates functions pivotal for cell growth, such as protein translation, glucose uptake, and glycolysis, and controls cell survival, through the phosphorylation and nuclear exclusion of transcription factors, which prevent the expression of genes, inducing cell death. AKT has several further targets, including the nuclear gene transcription mediators, e.g., forkhead box O (FOXO). RAS oncogenes are, instead, small guanosine 5′-triphosphate (GTP)-ases, which serve as master regulators of a myriad of signaling cascades involved in miscellaneous cellular processes. RAS transmits extracellular signals to intracellular effectors pathways: the activation of RAF kinases requires the interaction with RAS, which, in turn, activates mitogen/extracellular-signal-regulated kinases (MEK) and extracellular-signal-regulated kinases (ERK). Besides the so-called RAS/RAF/MEK/ERK cascade, better known as mitogen-activated protein kinase (MAPK), RAS can also regulate the PI3K/AKT/mTOR signaling pathway.

Resveratrol has effect on the phosphatase and tensin homolog (PTEN)/AKT pathway, commonly deregulated in prostate cancer. The direct effect of resveratrol corresponds to a reduction in phosphorylation of AKT, as well as of mTOR and FOXO; in particular, the dephosphorylation of FOXO results in its translocation to the nucleus and further activation (20). Consistently, a syngenic mouse model of melanoma showed as the treatment with resveratrol reduced tumor volume and metastasis throughout reduced AKT expression (21).

Therefore, these data support the specific activity of resveratrol in controlling the formation of multiprotein RPTKs complexes and related signaling pathways, i.e., AKT and mTOR, which, when impaired, are involved in all stages of carcinogenesis, from initiation to metastasis.

In addition, resveratrol directly inhibited all phases of carcinogenesis modulating red–ox signaling. Both antioxidant and prooxidant effects can have effect on carcinogenesis, despite the debate is still open regarding their relevance on chemoprevention. The antioxidant properties are assumed to protect cells from oxidative damage; however, prooxidant activities are held likely to be responsible for their cytotoxic or proapoptotic effects. As chemopreventive agent, resveratrol chronic supplementation reduced DNA leukocytic damage, altering the prooxidant/antioxidant balance, in a rat model of colon carcinogenesis induced by 1,2-dimethylhydrazine (22). The increase of antioxidant status occurred *via* both enzymatic mechanisms, involving superoxide dismutase (SOD), catalase (CAT), glutathione reductase, glutathione peroxidase, and glutathione *S*-transferase, as well as non-enzymatic mechanisms, associated with reduced glutathione, vitamin C, vitamin E, and β-carotene (22). A significant decrease in lipid peroxidation markers (thiobarbituric acid reactive substances, diene conjugates, and lipid hydroperoxides) was also reported (22). Conversely, as chemotherapeutics, resveratrol, used in combination with As_2_O_3_ against lung adenocarcinoma cells, enhanced apoptosis-inducing oxidative stress (23). In agreement, the effect of the resveratrol derivative, 3,3′,4,4′-tetrahydroxy-trans-stilbene, on viability, apoptosis, and proliferation of ovarian cancer cells included the influence on cell red–ox homeostasis (24). The antiapoptotic activity was associated with the cysteine-dependent aspartate specific proteases, namely, “caspases” (CASP) 3, 8, and 9, and p38 MAPK, which were stimulated more efficiently with the derivative than the parent drug (24). This effect coincided with increased reactive oxygen species (ROS) generation, downregulated SOD and CAT activities, excessive accumulation of 8-hydroxy-2′-deoxyguanosine, and its insufficient repair due to decreased expression of DNA glycosylase I (24). Moreover, the effects of resveratrol and its three derivatives, i.e., 3,3′,4,4′-tetrahydroxy-trans-stilbene (M6), 3,4,4′,5-tetrahydroxy-trans-stilbene (M8), and 3,3′,4,4′,5,5′-hexahydroxy-trans-stilbene (M12), showed strong cytotoxicity against T cell leukemia Jurkat cells (25). The increased activity of CASP 3 and 9 was again observed, particularly in presence of M8 and M12, short-living prooxidative metabolites; cell death was accompanied by the loss of mitochondrial potential, oxidative stress, decrease of glutathione levels as well as the loss of both mRNA expression and activity of manganese-dependent superoxide dismutase (MnSOD) (25).

The balance between antioxidant and prooxidant effects of resveratrol is still controversial, being dependent on the dosage in use for testing. Given the above reported results, two major and opposite functions of this phytochemical can be found: the first related to its chemoprevention activity associated with antioxidant effect in order to reduce impairment of damage of essential biomacromolecules; the second related to its chemotherapeutic potential, associated with the induction of ROS generation in cancer cells, leading to their enhanced apoptosis.

### Biological Outcomes of Growth Factor Signaling: Cell Proliferation and Genome Instability

Cell proliferation is the combination of two distinct processes: (i) cell cycle, including cell division and genome segregation (mitosis) and (ii) cell growth, under the influence of many growth factors. The four phases composing the mammalian cell cycle, i.e., S (DNA synthesis) and M (mitosis) separated by gap phases, G1 and G2, are mainly regulated by cyclin-dependent kinases (CDKs), and E3 ubiquitin ligases, which mediate targeted protein degradation. Several phytochemicals inhibit cancer cell proliferation by modulating the genes accounted to control several aspects of cell cycle, including the activity cyclins and CDKs. Cell cycle arrest is an irreversible process, which, ultimately, can result in the apoptotic cell death.

Resveratrol inhibited oral squamous cell carcinoma cell lines by inducing apoptosis and cell cycle arrest at G2/M transition, in a concentration- and time-dependent manner, enhancing the expression of cyclin A2 and cyclin B1 (26). Similar findings were reported for the resveratrol analog (E)-4-(3,5-dimethoxystyryl)phenyl acetate (Cmpd1), highly active against glioblastoma, one of the most malignant form of adult brain tumor with a very poor prognosis: Cmpd1 reduced glioblastoma cell colony formation, arresting cell cycle at G2/M phase transition and suppressing cyclin D1 and cyclin B1 expression (27).

Inducing the arrest at G1-phase, resveratrol resulted in a time- and dose-dependent inhibition A431 human epidermoid carcinoma cell growth, particularly by modulating the CDK inhibitor-cyclin-CDK machinery (28). Resveratrol treatment caused induction p21^Cip1^ (or p21^Waf1^), a CDK inhibitor 1 also called CDK-interacting protein 1, decreasing the protein expressions of cyclin D1, cyclin D2 and cyclin E as well as of CDK2, CDK4, and CDK6, which were also reduced in their activity (28). Analogous cycle arrest at G1-phase was also reported for liver carcinoma (29) and hepatoma (30) cell lines. To further support the findings on A431 cell line, Ahmad et al. also demonstrated the effect of resveratrol on retinoblastoma protein (pRb) and the E2F families of transcription factors, which are essential proteins for G1/S phase transition, mediating apoptotic cell death (31). Similar results were, then, reported for non-melanoma skin cancer cells by Cosco and colleagues (32). Authors provided evidence of the pRb-E2F/DP pathway as important contributor of resveratrol-mediated cell cycle arrest and apoptosis; indeed, resveratrol produced a dose- and time-dependent decrease in the hyperphosphorylated pRb with a relative increase in hypophosphorylated pRb, compromising the availability of free E2F and downregulating protein expression of five E2F (1–5) (31). The E2F factors drive cell to apoptosis, binding to DNA as homodimers or heterodimers in association with dimerization partner (DP) 1 and 2 (31).

The cell cycle arrest at S-phase and further apoptosis was observed after resveratrol treatment in anaplastic thyroid carcinoma cells, an extremely aggressive and undifferentiated tumor, for which conventional treatments are usually not effective (33). Resveratrol metabolites, i.e., resveratrol 3-*O*-d-glucuronide and resveratrol 4′-*O*-d-glucuronide (but not resveratrol 3-*O*-d-sulfate), showed similar effects in colon cancer cells, with G1/S phase arrest mediated by A3 adenosine receptors and cyclin D1 depletion (34).

The effects of resveratrol on cell cycle arrest and cell proliferation appeared to be also modulated by its specific targeting toward long non-coding RNAs (lncRNAs), which are transcripts longer than 200 nucleotides, currently considered the new avenue for chemoprevention. Changes in the expression of lncRNAs were reported in several forms of cancer and proposed as molecular target for phytochemicals. At microarray analysis, resveratrol downregulated the tumor-related lncRNA, called AK001796, in lung cancer cell line (35).

In summary, resveratrol has the function of reducing cell growth and inhibiting cell proliferation directly acting on cell cycle, growth factors, CDKs, and transcription factors. All together, these effects lead to the resveratrol-enhanced chemoprevention, by modulating genes involved in cancer cell initiation and proliferation as well as promoting cancer cell apoptosis.

Beyond cell growth, a further biological outcome of growth factor signaling is the genomic instability, major driving force for carcinogenesis since able to increase the tendency to accumulate genetic alterations, during the life cycle of cells. During cell division, genomic instability is usually minimized *via* high-fidelity DNA replication in S-phase, correct chromosome segregation in mitosis, and error repair of sporadic DNA damage. A well-documented anticancer mechanism of resveratrol works on blocking topoisomerase (TOPO) activity, a family of enzymes that regulate the over-winding or under-winding of DNA. Resveratrol, above all, interferes with TOPO II-α (TOPO2), involved in the resolution of super-coiled DNA and chromosome segregation during mitosis and over-expressed in cancer cells. Resveratrol, indeed, induced a delay in S-phase progression with the concomitant phosphorylation of the histone H2AX (H2A histone family, member X), which, in turn, inhibits TOPO2 (36, 37). Phase II metabolites of resveratrol displayed the same effects (38). Rusin and co-workers also demonstrated as resveratrol was associated with highly instable telomeres, phosphorylating the histone H2AX at serine 139 and p53 at serines 15 and 37, and activating DNA signaling (39), signs of senescence, including the impaired expressions of “BReast and ovarian CAncer growth-suppressor protein” (BRCA)-1, cyclin B1, pRb, and p21, were detectable, too (39). Nonetheless, resveratrol was associated with the prolongation of the TOPO–DNA complex, as reported for gliobastoma cells (40) and for breast and colon cancer cells, where the compound also correlated to lower nuclear levels of human telomerase reverse transcriptase (hTERT) (41). All together, these findings let resveratrol acquiring the role of “topoisomerase poison,” although data on osteosarcoma and lung cancers are still debated (39).

To date, resulting data support the role of resveratrol as chemotherapeutic agent, able to induce cell death specifically acting on TOPO2.

### Cytoplasmic Tyrosine Kinase Signaling

#### Cytokine Signaling

Several types of cancer are to some extent promoted by a certain degree of systemic, low-grade chronic inflammation, characterized by elevated circulating inflammatory biomarkers, such as cytokines (e.g., interleukin (IL)-8, IL-6, IL-1, and IL-12), prostaglandin E2 (PGE2), tumor necrosis factor α (TNF-α), and interferon (INF).

Resveratrol mainly acts on TNF-α expression and related signaling pathways. Noteworthy, considering human embryonic kidney cell line, resveratrol suppressed TNF-α-induced signaling in dose-dependent manner, both *via* nuclear factor kappa-B (NF-κB) activation and *via* transcriptional activity of p65, but without affecting the expression of the former or blocking the nuclear translocation of the latter (42). Resveratrol inhibited DNA-binding activity of NF-κB and IKKα degradation and blocked the ubiquitination of NF-κB essential modifier (NEMO), a subunit of the IκB kinase complex that activates NF-κB, but inhibits NF-κB kinase subunit gamma (IKK-γ) (42). In colon carcinoma cancer (CRC) cells, the downregulation of NF-κB activation and its translocation to the nucleus, as well as the downregulation of gene end products regulated by NF-κB, i.e., matrix metalloproteinase (MMP)-9 and CASP 3, were both mediated through the inhibition of NF-κB inhibitor α (IκBα) kinase (43).

A further involvement of the TNF-α signaling in the ­anticancer effect of resveratrol is associated with the radio- and chemoinducible cancer gene therapy vector “Ad.Egr-TNF,” a replication-deficient adenovirus expressing human TNF-α under control of the early growth response (Egr)-1 promoter (44). In rat tumor xenograft models, resveratrol induced the Egr-1 expression from its chromosomal locus [Egr-1 promoter CC(A + T) rich GG sequences], showing anticancer response with the increase of TNF-α (44). Interestingly, the inhibition of the nicotinamide adenine dinucleotide-dependent protein deacetylase, called sirtuin (SIRT)-1, a reported target of resveratrol, appeared to not abrogate the induction of Egr-1 expression, thus excluding a SIRT-mediated mechanism of action, in this case (44).

However, data support as SIRT1 decreases apoptosis through deacetylation of p53; resveratrol is known as an activator of SIRT1 (45) as well as of the gene expression levels of SIRT1, SIRT3, and SIRT4 as reported in the liver of wild-type zebrafish (46). Authors also documented resveratrol activity on peroxisome proliferator-activated receptor gamma coactivator 1-alpha (PGC1α), which is a transcriptional coactivator that regulates the genes involved in energy metabolism, and nicotinamide phosphoribosyltransferase (NAMPT), an enzyme which contributes to the biosynthesis of nicotinamide adenine dinucleotide, promoting vascular smooth muscle cell maturation, inhibiting neutrophil apoptosis, and activating insulin receptor (46). SIRT/NAMPT interacts with the acetylation of p53: using the 293T cell line (HEK293 cells transformed with large T antigen), the apoptosis induced by an inhibitor of NAMPT pathway was associated with increased acetylation of p53 at Lys382, which is required for the functional activity of p53 (47). Resveratrol, as SIRT1 activator, also ameliorated cisplatin-induced acetylation of p53, apoptosis, and cytotoxicity in mouse proximal tubular cells cells: SIRT1 activation by resveratrol decreased cisplatin-induced apoptosis while improving the glomerular filtration rate and resveratrol also remarkably blocked cisplatin-induced decrease of Bcl-xL (45). Taken together, findings suggest that the modulation of p53 by SIRT1 could be a possible target to attenuate cisplatin-induced kidney injury. Similarly, resveratrol alleviated doxorubicin (DOX)-induced myotoxicity (48) and cardiotoxicity (49) *via* SIRT1-dependent mechanisms.

The regulatory activity of resveratrol on sirtuins is also relevant in terms of antiageing effects. By regulating these histone deacetylase, resveratrol mimics caloric restriction, thus prolonging lifespan.

#### Integrin Signaling

Integrins are transmembrane proteins that facilitate the interaction of cells with the extracellular environment, strongly implicated in cancer progression. Transcriptional factors are the main elements influenced by integrins, although, in the last decades, a further integrin mediated process acquired more and more importance: the epithelial–mesenchymal transition (EMT), key player for tumor invasion and metastasis.

Considering the effect of resveratrol on transcriptional factors, besides the above described NF-κB (see Cytokine Signaling), resveratrol also inhibits several further transcriptional factors, including activator protein (AP)-1, AP-2, and CREB, which act, independently or in coordination, to control genes involved in regulating urinary plasminogen activator (u-PA) and several MMPs (35). When tested on osteosarcoma cells, resveratrol reduced their *in vitro* migration and invasion through transcriptional and epigenetic regulation of MMP-2; this finding was corroborated by *in vivo* experiments, which observed a decreased incidence of lung metastasis (35). From the molecular point of view, MMP-2 activity reduction was controlled *via* inhibition of CREB–DNA-binding activity and upregulation of miR-328, involving c-Jun N-terminal kinases (JNK) and p38 MAPK signaling pathways, respectively (35). Considering cancer suicide gene therapy for the treatment of lung adenocarcinoma, preclinical data support the use of resveratrol-responsive CArG domain elements [CC(A/T)6GG] from the Egr-1 promoter, in order to promote apoptosis and reduce the risk of metastasis (50). In lung adenocarcinoma cells, when combined with resveratrol, both synthetic and natural Egr-1 promoters induced the expression of the exogenous (but not the endogenous) nuclear protein called “growth arrest and DNA damage” (GADD)-45α, particularly responsive to genomic instability finally promoting apoptosis *via* ERK/JNK-ElK-1 pathway (50). Resveratrol-induced reduction of cell invasion was also observed and associated with a decreased expression of AP-1/JunD, MMP-1, B-cell lymphoma (Bcl)-2, inducible NOS (iNOS), and those molecules related to alpha-melanocyte-stimulation hormone (α-MSH) signaling, e.g., β-catenin, c-kit, and microphthalmia transcription factor (MITF) (51).

Currently, the overwhelming activity of resveratrol on integrin signaling has been associated with the arrest of EMT, opening innovative ways to treat highly metastatic and life-threatening tumors, such as the pancreatic adenocarcinoma. Resveratrol inhibited, in a dose-dependent manner, EMT of pancreatic cancer cells, by suppressing both the PI3K/AKT/NF-κB pathway and the EMT-related gene expression (E-cadherin, N-cadherin, vimentin, MMP-2, and MMP-9), pivotal for cancer cell motility and metastasis (52, 53). Consistently, resveratrol reduced the levels of phospho-AKT and phospho-NF-κB in pancreatic cancer cells, and counteracted the alterations of cell morphology, typical of EMT and cell invasion, induced by the transforming growth factor-β (TGF-β) (53). Along the same direction, resveratrol decreased the levels of cell adhesion proteins and EMT mediators: α5β1 integrins and hyaluronic acid were repressed in ovarian cancer cell lines (54); N- and E-cadherins and the protein able to promote their suppression, i.e., Snail family Zinc finger 1 (SNAI1, also called Snail), were decreased in gastric (55) and CRC cells (56). Resveratrol, moreover, potentiated the antitumor effects of 5-fluoruracyl (5-FLU) on CRC cells enhancing their chemosensitization and attenuating the drug resistance: the mechanism involved the suppression of EMT phenotype, with the upregulation of intercellular junctions and E-cadherin, and the downregulation of NF-κB and vimentin (43).

#### Developmental Signaling Pathways

Multiple cellular developmental signaling pathways have been involved in the processes of cancer cell invasion, metastasis, and tumor relapse. Wingless integration (Wnt), Notch, Hedgehog (Hh), and signal transducer and activator of transcription (STAT3) are crucially implicated in the embryonic development, in the biology of cancer stem cells (CSCs) and in the acquisition of EMT. Resveratrol, in particular, has been reported to act mostly on STAT3 (57) and Notch signaling (33). Zhong et al. reported that resveratrol reduced gene activation and protein expression of STAT3, Notch, and Wnt signaling in ovarian cancers, enhancing G1 phase accumulation and increasing apoptosis (53). Again, in cervical adenocarcinoma and squamous cell carcinoma cell line, resveratrol inhibited cell growth by promoting apoptosis *via* suppression of STAT3, Notch1, and Wnt2 signaling pathways (58). Considering human biopsies of cervical cancer, the immunohistochemical staining on tissue microarrays revealed significantly higher frequencies of Notch1, Notch2, Hes1, Wnt2, Wnt5a, p-STAT3, and β-catenin nuclear translocation than in healthy controls (58). On the other hand, the protein inhibitor of activated STAT3 (PIAS3), which is an E3 small ubiquitin-related modifier (SUMO)-protein ligase, was remarkably low in tumor specimens (58). In agreement with these findings, resveratrol suppressed the Wnt signaling in both normal and tumor colon mucosa in a phase I pilot trial on eight colon cancer patients (57) and also inhibited the Notch1 pathway in anaplastic thyroid carcinoma cell line, following a dose-dependent manner (33). In the latter case, resveratrol upregulated the expression of thyroid-specific genes, including thyroid transcription factor (TTF)-1 and TTF-2, paired box (Pax)-8, and sodium iodide symporter, inducing functional Notch1 protein expression and activation of transcriptional factors related to this pathway (33). Similarly, resveratrol promoted the apoptosis in glioblastoma cells *via* the wild-type p53 restoration, dependent from the Notch1 signaling (59).

The resveratrol analog Cmpd1 further support the role of STAT3 in anticancer molecular mechanism of this phytochemical: glioblastoma cells, in particular, harbor aberrantly active STAT3 and the treatment with Cmpd1 suppressed the STAT3 tyrosine705 phosphorylation in a dose-dependent manner, concomitantly with the induction of the STAT3 pserine727 and Erk1/2 (27). Cmpd1 also blocked EGF-stimulated pSTAT1 induction (27).

Besides STAT3, Notch, and Wnt, the Hedgehog (Hh) signaling pathway plays a pivotal role in carcinogenesis and, specifically, in EMT promotion, representing an important target for resveratrol. In gastric cancer, resveratrol reduced the expressions of glioblastoma (GL)-1, a protein originally isolated in human glioblastoma and key effectors of the Hh pathway during embryo development and upregulated in many tumors (55). Decreasing the levels of crucial integrins for EMT, such as Snail, E-cadherin, and N-cadherin, resveratrol suppressed the cell viability and invasion similarly to what occurred in presence of cyclopamine (55). The phytochemical also reduced mRNA and protein levels of the following Hh signals: the Indian hedgehog homolog (Ihh) and the surface transmembrane protein, called Patched (Ptch), which prevents the high expression of the seven membrane spanning receptor, namely, smoothened (SMO) (56). Resveratrol, finally, regulated the expression of protein kinase C (PKC) α and δ, in a time- and dose-dependent manner, similarly to 5-FLU used as positive control (56).

#### Signal Transduction by the Transforming Growth Factor-β Super-Family

TGF*-*β super-family has been widely implicated in various cellular processes, including cell proliferation and differentiation, immune modulation, and extracellular matrix remodeling. TGF*-*β exhibits antiproliferative functions by activating signaling pathways that mediates cell cycle arrest and induction of apoptosis. It also exerts effects through heteromeric receptor complexes consisting of type I and type II serine/threonine kinase receptors. The signaling pathway is initiated by the ligand binding to the TGF*-*β RII cell surface receptor, which, in turn, recruits the TGF*-*β RI kinase. The latter phosphorylates the receptor-regulated son of mothers against decapentaplegic (R-SMAD) proteins, namely, Smad2 and Smad3. Activated R-Smads form a complex with the common-mediator Smad (Co-Smad), called Smad4, which then moves directly to the nucleus. The complex can either act as a co-activator or DNA-binding transcription factor, participating in the regulation of several target gene expression (e.g., p15, p21, and c-myc). TGF*-*β is further controlled by a third class of Smads, the inhibitory Smads (i-Smads), named Smad6 and Smad7 proteins, which block the activation of R-SMADs and Co-SMADs. TGF-β signaling, at later stages, can induce EMT.

Resveratrol, particularly, reduced the alterations of cell morphology induced by TGF-β, typical of EMT and cell invasivity, *via* AKT and NF-κB (53). Similar findings were reported for CRC *in vitro* and *in vivo*: the expression of E-cadherin, *via* transcription factors Snail, was increased, while vimentin expression and the activation of the TGF-β1/Smads signaling pathway were reduced (60).

#### Apoptotic Pathways

Programed cell death, or apoptosis, is a highly conserved physiological cell suicide response, crucial for mammalian tissue homeostasis. Cell apoptosis is triggered in response to endogenous stimuli (e.g., growth factor deprivation) or exogenous stimuli (e.g., irradiation or genotoxic chemotherapeutic drugs). Mammalian apoptotic pathways are greatly controlled by a plethora of molecular mechanisms, which involve both antiapoptotic proteins, such as Bcl-2, and proapoptotic proteins, such as CASPs and FAS ligand (FAS-L). Over-expression of Bcl-2 family proteins inhibits the release of cytochrome *c* from the mitochondrial intermembrane space into the cytosol, which is a decisive event triggering apoptosis. CASPs possess a wide range of expression patterns throughout mammalian tissues and are usually organized into a cascade of proteins: some CASPs are “initiators” (CASP-8, 9, and 10) and sequentially process and activate other CASPs, called “executioners” (CASP-3, 6, and 7). The FAS receptor (FasR) is a surface death receptor able to bind its specific ligand to lead cell toward apoptosis: it is also known as apoptosis antigen 1 (APO-1 or APT), cluster of differentiation 95 (CD95), or TNF-receptor super-family member 6 (TNF-R-SF6). After binding, the death domain (DD) aggregation produces the receptor complex internalization *via* endosomes; this allows the adaptor molecule to bind the DD of Fas through its own DD, resulting in the formation of Fas-associated protein with death domain (FADD). FADD also contains death effectors domain, which facilitates binding to CASP-8.

As demonstrated on prostate cancer cells, the molecular mechanisms of resveratrol exploit the interactive effects with TNF-related apoptosis-inducing ligand (TRAIL), a protein that works as a ligand and induces the process of programed cell death. Prostate cancer androgen-dependent LNCaP cells, in particular, are resistant to TRAIL, and the downregulation of PI3K/AKT pathway by resveratrol can sensitize cells toward TRAIL-mediated apoptosis. Treatment of LNCaP cells with resveratrol resulted in generation of ROS; translocation to mitochondria of both Bcl-2-like protein 4, also called Bcl-2-associated X protein (Bax), and p53 tumor suppressor protein, with the subsequent drop in mitochondrial membrane potential. Released mitochondrial proapoptotic proteins include, among the oth­ers, cytochrome *c* CASP-3 and CASP-9, apoptosis-inducing factor (AIF), second mitochondria-derived activator of caspase/direct inhibitor of apoptosis-binding protein with low pI (Smac/DIABLO) and protein high temperature requirement serine protease A2 (HtrA2), also known as Omi (61, 62). Smac was, in particular, investigated and authors found that Smac small interference RNA (siRNA) inhibited resveratrol-induced apoptosis, whereas Smac N7 peptide induced apoptosis, enhancing the effectiveness of resveratrol (61, 62). In the same work, resveratrol also augmented the expression of further proapoptotic proteins, i.e., Bax, Bcl-2 -antagonist/killer-1 (Bak), p53 upregulated modulator of apoptosis (PUMA), the Bcl-2 Homolog (BH)3-containing proteins (called Noxa and Bim), TRAIL death receptors – R1/DR4 and R2/DR5 (61, 62). Conversely, resveratrol downregulated the expression of antiapoptotic proteins, including Bcl-2, Bcl-XL, survivin, and XIAP (61, 62) Resveratrol had no effect, instead, on normal human prostate epithelial cells and also the ability of the phytochemical to sensitize TRAIL-resistant LNCaP cells was inhibited by the presence of dominant negative FADD, CASP-8 siRNA, or *N*-acetylcysteine (61, 62). Similar anticancer effects of resveratrol was observed on fibrosarcoma cells, where the expression of apoptosis-associated genes resulted altered in microarray analyses (63), as well as on nasopharyngeal cancer cells and non-small cell lung cancer cells, where resveratrol inhibited cell viability and promoted apoptosis by activating citocrome *c* (64), CASP-3 and altering the Bax/Bcl-2 apoptotic signaling (65).

Again, the resveratrol derivative, 3,3′,4,4′-tetrahydroxy-trans-stilbene, and the analog cmpd1 stimulated CASP activity (3, 8, and 9) in ovarian cancer cells (24), glioblastoma cells (27) and ovarian cells (17); in the latter two cases, resveratrol also induced cleavage of poly(ADP ribose) (PARP-1) (17) and suppressed survivin, myeloid cell leukemia 1, B-cell lymphoma-extra-large (Bcl-xL) (27). In Huh-7 cells, a human hepatoma cell line system infected by hepatitis C virus, resveratrol promoted the expression of proapoptotic proteins, which was associated with the mitochondrial membrane depolarization and the increase in CASP activity (30). However, the phytochemical had no effect on death receptor Fas, FasL, ERK ½, and p38 expression, while downregulated phospho-ERK and phospho-p38 expression (30), triggering autophagic cell death through increased expression of autophagy-related genes (Atg)-5, Atg7, Atg9, and Atg12 (30). Consistently, the antiproliferative activity of resveratrol was reported also in two human liver cancer cell lines, Hep p53-positive G2 and p53-negative Hep 3B. Resveratrol inhibited cell growth in p53-positive Hep G2 cells only, as a result of apoptotic cell death *via* p53-dependent pathways (29). These cells, arrested at G1 phase, showed the increase of p21 and Bax expression, but not of Fas/APO-1 apoptotic signaling pathway (29). Conversely, when tested in combination with As_2_O_3_, resveratrol synergistically increased not only the release of cytochrome *c*, but also the expressions Fas and FasL, and the apoptotic pathways mediated by oxidative stress (23).

In addition to p53 pathway, resveratrol contributed to trigger apoptosis *via* pRb-E2F/DP, as demonstrated in A431 human epidermoid carcinoma cells (31).

These findings identify resveratrol as a powerful proapoptotic agent, which is able to trigger different pathways leading to both mitochondrial and non-mitochondrial programed cell death, in a number of cancer cells. Noteworthy, the stilbene can sensitize resistant cancer cells without significantly affecting normal (non-cancer) cells.

### Inflammatory Pathway

Inflammation is a critical component of tumor progression with a key role within the tumor microenvironment. Signaling molecules of the innate immune system are, indeed, involved in cancer invasion, migration, and metastasis, such as selectins, chemokines, and their receptors. These observations could be used for new inflammatory therapeutic approaches against cancer development that could involve inflammatory agents, from non-steroidal inflammatory drugs (NSAIDs), selective cyclooxygenase-2 (COX-2) inhibitors to natural products, mainly phytochemicals.

Resveratrol is a promising molecule able to target multiple inflammatory, cancer-related sites, simultaneously, i.e., macrophage migration inhibitory factor, COX-2, NF-κB, and AP-1 (15, 66, 67). In particular, this compound is a strong COX suppressor, and an activator of peroxisome proliferator-activated receptor (PPAR) (68). Recent evidence supports the role of microRNAs (miRNAs) in the beneficial effects of resveratrol, with emphasis on its inflammatory effects: it decreased the secretion of pro-inflammatory cytokines (e.g., IL-6, IL-8, and TNF-α), the expression of adhesion proteins, such as intercellular adhesion molecule (ICAM)-1, and of leukocyte chemoattractants, including monocyte chemoattractant protein (MCP)-1, and it increased the production of inflammatory cytokines (67). Although without a clear mechanistic link, resveratrol activity appeared to be partially dependent from an impaired expression of certain miRNAs associated with inflammatory and tumor suppression effects (miR-663), proinflammatory effects (miR-155), or oncogenicity (miR-21).

To date, however, evidence is particularly sound in sustaining the anti-inflammatory activity of resveratrol, over the others natural molecules, to counteract the proliferation of CRC (69) and MCF-7 breast cancer cell line, *via* p53-COX-2 pathway (70).

Inflammation milieu is pivotal in cancer development, particularly in chronic inflammatory conditions. Resveratrol anti-inflammatory activity is well-documented in a number of cancer cells and it is based on different mechanisms, thus contributing to explain the plethora of anticancer pathways synergistically regulated by this polyphenol.

### Immune Surveillance

In the last decades, several molecular mechanisms related to the immune activation have been demonstrated to protect against the onset of tumor cells, supporting the immunotherapy against cancer. The dysfunction of the host’s immune system, conversely, is associated with the suppression of tumor immunosurveillance and, in particular, T cell anergy, the presence of regulatory T cells and systemic defects of dendritic cells have been reported to be the main responsible factors of immune evasion. Immunosurveillance suppression involves resistance to apoptosis, secretion of immunosuppressive cytokines, reduced expression of major histocompatibility complex class I antigens and immunomodulatory molecules. Both host- and tumor-related mechanisms can lead to a progressive failure of the tumor-specific immune response, limiting the success of cancer immunotherapy.

Macrophages, particularly, inhibited or promoted the growth and spread of cancer, depending on their activation state. Treatment with the synthetic resveratrol analog HS-1793 significantly increased IFN-γ secreting cells in splenocytes, and also decreased CD206+ macrophage infiltration, compared to CD68+ cells, in those tumor sites with a higher expression of IFN-γ (71). The local increase of IFN-γ modulated the status of tumor-associated macrophages (TAM) associated with the cancer microenvironment: human monocytic cell line THP-1 cells, stimulated with phorbol-12-myristate-13-acetate, differentiated to macrophages with M2-like phenotypes, which display TAM-like properties (71). The latter include high level of CD206, CD204, IL-10, TGF-β, EGF, MMP-9 and low levels of IL-6 and IL-12, able to promote tumor cell invasion (71). Upon IFN-γ exposure, THP-1-derived TAM changed their phenotypes to M-1-like morphology and intracellular granular pattern, showing increased levels of pro-inflammatory and immunostimulatory cytokines, and a reduction in immunosuppressive and tumor progressive mediators (71).

Evidence on the effects of resveratrol on cancer immunesurveillance is still scanty, and more mechanistic data are needed with non-synthetic resveratrol derivatives.

### Hormone Signaling

The estrogenic activity has been recently reported for resveratrol and for its blood-circulating metabolites, applying a yeast two-hybrid detection system, which relies on the interaction between the ligand-binding domain of the human estrogen receptors (ERs) α and β and the human co-activator transcriptional intermediary factor (Tif)-2 (72). Only the metabolite resveratrol-3-*O*-sulfate displayed strong, estrogenic activity *via* ERα-preferential antagonistic pathway, as confirmed in a human breast adenocarcinoma cell line (72). Consistently, the presence of 17-beta-estradiol further enhanced MAPK activation induced by resveratrol, but blocked the resveratrol-enhanced apoptosis, *via* inhibiting the p53-directed transcriptional activity, the p53 phosphorylation at serines 15, 20, and 392, and the p53 acetylation, in a concentration- and time-dependent manner (73). All these effects were arrested by the inhibitor of the nuclear ERα (73).

Beyond estrogenic activity, the androgenic effect of resveratrol represents an important step toward novel anticancer strategies. Dihydrotestosterone (DHT) can promote breast cancer growth *via* several mechanisms and, in addition to binding to ERα, the DHT binds its specific membrane receptor on integrin αvβ3. Interestingly, resveratrol can bind the plasma membrane integrin αvβ3, too, but it induces p53-dependent apoptosis. Therefore, resveratrol and DHT are both transducer, following the pathway of activated MAPK ERK1/2, but DHT enhances cell proliferation in cancer cells, while resveratrol promotes apoptosis (70). The mechanism of such diametrically opposed effect is dependent by two distinct types of ERK1/2 activation, each one associated with specific serine phosphorylation and acetylation of p53, mediated by separate surface receptor sites (70). In MCF-7 breast cancer, as a result, DHT inhibited several resveratrol-stimulated effects: (i) phosphorylation of Ser-15 of p53, (ii) COX-2/p53-dependent gene expression and nuclear complex of p53–COX-2 formation, essential for p53-dependent apoptosis, and (iii) p53-directed transcriptional activity (70).

In terms of chemoprevention, however, recent clinical findings do not support the use of resveratrol in the treatment of benign prostate hyperplasia (74). In a randomized placebo controlled clinical trial, two doses of resveratrol (150 or 1,000 mg, daily), administered for 4 months to 66 middle-aged men suffering from the metabolic syndrome, had no effects on prostate size and levels of prostate-specific antigen (PSA), testosterone, free testosterone and DHT (74). The highest dose of resveratrol, eventually, lowered the serum level of androstenedione, dehydroepiandrosterone (DHEA), and, mainly, dehydroepiandrosterone sulfate (DHEAS) (74), without a clear clinical significance.

Therefore, as reported above, resveratrol can target hormone signaling due to its anti-estrogenic activity relevant in hormone-dependent cancers. However, the effects of this phytoestrogen on the androgen pathway should be further ascertained.

### Multidrug Resistance

Cancer cells contain multiple aberrant signaling pathways that lead to drug resistance and therapy failure in many patients; thus, the multidrug therapy can possess higher efficacy against cancer cells than traditional therapies, by acting on diverse mechanisms simultaneously. As an example, the activated STAT3 signaling, above described as critical molecular target of resveratrol, appears a promising candidate in the management of resistant ovarian cancers (53). Considering gene therapy, the control of transgenic expression, *via* resveratrol activation, of Egr-1 promoter may sensitize cancer cells, extending the use of Ad.Egr-TNF to patients intolerant of radiation or chemotherapy, offering a novel tool for development of inducible gene treatments (44). Since these approaches are still seminal, resveratrol has been mostly investigated as adjuvant agent to be used in combination with classical chemotherapeutics, in the perspective of avoiding or reducing the risk of MDR.

Currently, resveratrol has been proposed as useful for anticancer therapy in combination with cisplatin (CIS) (64) and 5-FLU (32, 43), but not with DOX (38).

Resveratrol blocked the proliferation of both parental CRC cell lines (HCT116 and SW480) and their corresponding isogenic 5-FLU-chemoresistant derived clones (HCT116R and SW480R), synergizing the inhibitory effects of 5-FLU against cell invasion (43). Interestingly, resveratrol induced a transition from 5-FLU-induced formation of microvilli to a planar cell surface, concomitantly with the upregulation of desmosomes, gap- and tight junctions (claudin-2), and E-cadherin in both types of CRC cells (43). Along this direction, resveratrol- and 5-FLU were loaded together on ultra-deformable liposomes, in order to increase their skin permeation: the perspective accounted the use of these agents to treat squamous cell carcinoma, actinic keratosis, Bowen’s disease, and keratoacanthoma (32). Strongly arresting cell proliferation at G1/S phase, the multidrug liposomes improved the anticancer activity on human non-melanoma skin cancer cells as compared to both the free drug forms and the single entrapped agents (32). The ultra-deformable liposomes could accumulate in deeper skin layers and generate a skin depot of drugs, from which resveratrol and 5-FLU were slowly released (32).

The anticancer activity of CIS in combination with resveratrol has been tested in non-small cell lung cancer, in a dose- and time-dependent manner (64). The increased apoptosis was associated with the depolarization of mitochondrial membrane potential, the release of cytochrome *c* from mitochondria to cytosol, and the abnormal expression of Bcl-2 and Bax proteins (64).

In combination with DOX, resveratrol displayed the interference at the target enzymes, since both DOX and resveratrol act as TOPO inhibitors. In colon carcinoma cells, resveratrol, actually, counteracted DOX-induced formation of DNA-TOPO2 intermediates (38). At high concentrations (≥200 μM), resveratrol even diminished the intracellular concentrations of DOX, modulating its cytotoxic activity (38).

Resveratrol was added to arsenic trioxide (As_2_O_3_), a potent anticancer drug used for the treatment of acute promyelocytic leukemia and lung tumor, though largely compromised by MDR. In lung adenocarcinoma A549 cells, resveratrol potentiated the toxicity of As_2_O_3_, *via* apoptotic pathway (23). Resveratrol and As_2_O_3_ caused more genotoxicity and oxidative stress than the single agent, synergistically increasing the release of cytochrome *c*, the expressions of Fas and FasL, and apoptotic cell death *via* the induction of oxidative stress (23).

The chemosensitization of cancer cells to conventional chemotherapeutic agents represents an exciting field. In these terms, resveratrol represents a promising sensitizing agent in adjuvant therapy. Further studies should also ascertain its role in reverting radioresistance and in reducing the adverse effects of conventional therapies on non-target cells and tissues.

## Conclusion

Resveratrol appears a promising agent, relevant for both cancer chemoprevention and treatment. Several signaling pathways, involved in carcinogenesis and host defense response, are targets of its mechanism of action and mediate its pleiotropic anticancer activity. In the adjuvant therapy, in particular, resveratrol showed synergistic effects with 5-FLU and CIS chemotherapeutics, thus increasing cancer cell sensitization. The final perspectives are reducing adverse effects of conventional therapies to non-target cells and tissues, decreasing the risk of MDR and enhancing the global efficacy of treatments.

## Author Contributions

EV and MI conceived and designed the work, prepared the figures, and wrote the draft; AF and JS-R performed the data search and analysis, the figure and table preparation, and revised the draft.

## Conflict of Interest Statement

The authors declare that the research was conducted in the absence of any commercial or financial relationships that could be construed as a potential conflict of interest.

## Abbreviations

α-MSH, alpha-melanocyte-stimulation hormone; 5-FLU, 5-fluoruracyl; AIF, apoptosis-inducing factor; AP, activator protein; APO-1 or APT, apoptosis antigen 1; Atg, autophagy-related genes; Bak, Bcl-2-antagonist/killer-1; Bax, Bcl-2-associated X protein; Bcl, B-cell lymphoma; Bcl-xL, B cell lymphoma-extra large; (BH)3, Bcl-2 homolog; BRCA, BReast and ovarian CAncer growth-suppressor protein; CASP, caspases; CAT, catalase; CD95, cluster of differentiation 95; CDKs, cyclin-dependent kinases; cGMP/PKG, cyclic guanosine monophosphate/cGMP-dependent protein kinase; Cmpd1, (E)-4-(3,5-dimethoxystyryl)phenyl acetate; COX-2, selective cyclooxygenase-2; CRC, colon carcinoma cancer; CREB, cAMP response element binding; CSCs, cancer stem cells; DAG, diacylglycerol; DD, death domain; DHEA, dehydroepiandrosterone; DHEAS, dehydroepiandrosterone sulfate; DHT, dihydrotestosterone; DP, dimerization partner; EGF, epidermal growth factor; EGF-R, epidermal growth factor receptor; Egr, early growth response; EMT, epithelial–mesenchymal transition; ERs, estrogen receptors; ERK, extracellular-signal-regulated kinases; SNAI1 or Snail, family zinc finger 1; FADD, Fas-associated protein with death domain; FasL, Fas ligand; FasR, Fas receptor; FOXO, forkhead box O; GADD, growth arrest and DNA damage; GL1, glioblastoma; GTP, guanosine 5′-triphosphate; H2AX, H2A histone family, member X; Hh, Hedgehog; hTERT, human telomerase reverse transcriptase; HtrA2, high temperature requirement serine protease A2; HUVECs, human umbilical vein endothelial cells; IAPs, baculoviral inhibitor of apoptosis proteins; ICAM, intercellular adhesion molecule; IκBα, inhibitor of nuclear factor kappa B (NF-κB) α; NF-κB inhibitor α; Ihh, Indian hedgehog homolog; IKK, kinase subunit; IL, interleukin; INF, interferon; JNK, c-Jun N-terminal kinases; lncRNAs, long non-coding RNAs; M12, 3,3′,4,4′,5,5′-hexahydroxy-trans-stilbene; M6, 3,3′,4,4′-tetrahydroxy-trans-stilbene; M8, 3,4,4′,5-tetrahydroxy-trans-stilbene; MAPK, mitogen-activated protein kinase; MCP, monocyte chemoattractant protein; MDR, multidrug resistance; MEK, mitogen-activated protein kinase kinase; miRNAs, microRNAs; MMP, matrix metalloproteinase; mTOR, mammalian target of rapamycin; NAMPT, nicotinamide phosphoribosyltransferase; NEMO, NF-κB essential modifier; NF-κB, nuclear factor kappa-B; NO, nitric oxide; NOS, NO synthase; NSAIDs, non-steroidal inflammatory drugs; Omi, protein HtrA2; u-PA, plasminogen activator; PARP-1, poly(ADP ribose); Pax, paired box; PDK, phosphoinositide-dependent kinase; PGC1α, proliferator-activated receptor gamma coactivator 1-alpha; PGE2, prostaglandin E2; PI3K, phosphatidylinositol 3-kinase; PIAS3, protein inhibitor of activated STAT3; PKB or AKT, protein kinase B; PKC, protein kinase C; PPAR, peroxisome proliferator-activated receptor; PSA, prostate-specific antigen; Ptch, patched; PTEN, phosphatase and tensin homolog; PUMA, p53 upregulated modulator of apoptosis; RAF, rapidly accelerated fibrosarcoma; RAS, rat sarcoma; ROS, reactive oxygen species; RPTKs, receptor protein tyrosine kinases; pRb, retinoblastoma protein; Smac, second mitochondria-derived activator of caspase; Smac/DIABLO, second mitochondria-derived activator of caspase/direct inhibitor of apoptosis-binding protein with low pI; siRNA, small interference RNA; SIRT, sirtuin; Smad, son of mothers against decapentaplegic; SMO, smoothened; STAT3, signal transducer and activator of transcription; SUMO, small ubiquitin-related modifier; SOD, superoxide dismutase; MnSOD, manganese-dependent superoxide dismutase; TGF-β, transforming growth factor-β; TAM, tumor-associated macrophages; Tif, transcriptional intermediary factor; TNF-α, tumor necrosis factor α; TNFRSF6, TNF-receptor super-family member 6; TOPO, topoisomerase; TORC, transducer of regulated CREB; TRAIL, TNF-related apoptosis-inducing ligand; TTF, thyroid transcription factor; VEGF, vascular endothelial growth factor; Wnt, wingless integration; XIAP, X-linked inhibitor of apoptosis.
